# Identification roles of NFE2L3 in digestive system cancers

**DOI:** 10.1007/s00432-024-05656-y

**Published:** 2024-03-22

**Authors:** Fan Li, Zhili Wen

**Affiliations:** Department of Gastroenterology, The Second Affiliated Hospital, JiangXi Medical College, Nanchang University, Nanchang, China

**Keywords:** NFE2L3, Digestive system cancers, Biomarker, Diagnosis, Prognosis

## Abstract

**Background:**

Morbidity and mortality rates of Digestive System Cancers (DSC) continue to pose human lives and health. Nuclear factor erythroid 2-like protein 3 (NFE2L3) is aberrantly expressed in DSC. This study aimed to explore the clinical value and underlying mechanisms of NFE2L3 as a novel biomarker in DSC.

**Methods:**

We utilized data from databases and clinical gastric cancer specimens to validate the aberrant expression level of NFE2L3 and further assessed the clinical value of NFE2L3. To investigate the potential molecular mechanism of NFE2L3, we analyzed the correlation of NFE2L3 with immune molecular mechanisms, constructed PPI network, performed GO analysis and KEGG analysis, and finally explored the biological function of NFE2L3 in gastric cancer cells.

**Results:**

NFE2L3 expression is up-regulated in DSC and has both prognostic and diagnostic value. NFE2L3 correlates with various immune mechanisms, PPI network suggests proteins interacting with NFE2L3, GSEA analysis suggests potential molecular mechanisms for NFE2L3 to play a role in cancer promotion, and in vitro cellular experiments also confirmed the effect of NFE2L3 on the biological function of gastric cancer cells.

**Conclusion:**

Our study confirms the aberrant expression and molecular mechanisms of NFE2L3 in DSC, indicating that NFE2L3 could serve as a novel biomarker for diagnosis and prognosis of DSC.

## Introduction

Globally, tumors continue to pose a threat to human life and health. In 2021, statistics from the American Cancer Society showed that there were 338,090 new cases and 169,280 deaths were estimated for digestive tract tumors in the United States (Siegel et al. 2021). DSC mainly include cholangiocarcinoma (CHOL), colon adenocarcinoma (COAD), esophageal carcinoma (ESCA), liver hepatocellular carcinoma (LIHC), pancreatic adenocarcinoma (PAAD), rectum adenocarcinoma (READ), and stomach adenocarcinoma (STAD), continue to be major types of cancer threatening human health and survival. DSC are affected by several risk factors, including lifestyle habits, tobacco use, alcoholism, physical activity, and gender (Xie et al. 2021). Therefore, it is of great importance to identify a biological target with ideal diagnostic and therapeutic importance for patients with tumors.

NFE2L3 was discovered in the 1990s, belonging to the cap 'n' collar basic-region leucine zipper family, mainly located in the endoplasmic reticulum and the nuclear envelope Chevillard and Blank (2011). Currently, researches related to ap 'n' collar basic-region leucine zipper family primarily focus on nuclear factor erythroid-derived 2-like 2 (NFE2L2). NFE2L2 plays the role of the primary transcriptional regulator for the cell's antioxidant response controls, which are associated with cancer and drug resistance. The binding of kelch-like ECH-associated protein 1 (KEAP1) to NFE2L2 causes polyubiquitylation, leading to swift degradation of NFE2L2 and significantly influencing the initiation, progression, and evolution of tumors. The mechanism of NFE2L3 in cancers is entirely different from NRF2, but limited research has evaluated the significance of NFE2L3 as an innovative indicator in malignancies.

NFE2L3, as a crucial regulator of the cellular stress response, expressed in diverse tissues of the human, different levels of expression highlight the unique functionality of NFE2L3 in different tissues (Qian et al. 2022; Chen et al. 2019; Zhang et al. 2022). In recent years, a growing number of evidence has indicated the participation of NFE2L3 in the processes of differentiation, inflammation, and carcinogenesis. James Saliba demonstrated that the deletion of NFE2L3 results in decreased inflammation and a shift in the tumor microenvironment via the IL33 and RAB pathways (Saliba et al. 2022). Knockdown of NFE2L3 inhibited bladder cancer cell proliferation by inducing the cell cycle and apoptosis (Qian et al. 2022). In vitro and in vivo experiments support NFE2L3 as an oncogene involved in the process of tumor formation. Although findings have suggested that NFE2L3 may be a crucial regulator of cancer progression, the underlying molecular mechanisms remain poorly understood.

The prevalent approach to treating cancer involves a combination of chemotherapy, radiotherapy, and surgical excision of the tumor. Tumor immunotherapy as a novel therapeutic modality has revolutionized cancer treatment (Galluzzi et al. 2020; Wang et al. 2020; Feng et al. 2021). The investigators discovered that NFE2 family is a potential prognostic biomarker and is associated with immune infiltration in many cancers (Ju et al. 2020).

The goal of the study was to investigate the possibility of NFE2L3 as novel biomarker for DSC. We import data from different database to provide a systematic and comprehensive insight into NFEE2L3 expression level, diagnostic and prognostic value, and underling mechanism in DSC. We also used patients’ specimens and in vivo models test to confirm the biological functions of NFE2L3 in DSC.

## Materials and methods

### Gene expression analysis

All raw data were downloaded from The Cancer Genome Atlas (TCGA), University of California Santa Cruz (UCSC) Xena browser (https://xena.ucsc.edu/), and the Human Protein Atlas (HPA) (https://www.proteinatlas.org/). Raw data were processed using Strawberry Perl 5.30.0.1 and R 4.1.1. The Wilcoxon test was used to analyze the data. *p* < 0.05 (*) was considered statistically significant.

Twelve gastric cancer and normal tissues from patients admitted to Second Affiliated Hospital of Nanchang University. Written informed consent was obtained from the patients. This study was conducted in accordance with the guidelines of the Declaration of Helsinki, and approved by the Medical Ethics Committee of the Second Affiliated Hospital of Nanchang University (No. 2022096 Approve data: 2022.9.7). The recruitment period for this study from October 1, 2022 to January 30, 2023.

### Tumor mutational burden and microsatellite instability of NFE2L3 in pan-cancer

We extracted relevant data of Tumor mutation burden (TMB) and Microsatellite Instability (MSI) from raw data, and the relationship between NFE2L3 and pan-cancer was analyzed using Spearman’s rank correlation coefficient.

### Clinical relevance of NEF2L3 in DSC

We created receiver operating characteristic (ROC) curves using the R package “pROC” to examine the diagnostic value of NEF2L3 in DSC. The ROC curve area (AUC) between 0.7 and 0.9 indicated sure precision, and the AUC over 0.9 indicated great accuracy. The prognostic effect of NFE2L3 in DSC was investigated by performing Kaplan–Meier survival analysis. We selected three markers for the Kaplan–Meier analysis: overall survival (OS), disease-specific survival (DSS), progression-free interval (PFI). Both the “survival” and “survminer” packages were used to plot the survival curves. The relationship between NFE2L3 expression and clinical features is studied using “limma” and “ggpubr” packages.

### NFE2L3 expression in subtypes and immune infiltration

NFE2L3 expression in immunological or molecular subtypes was measured in TISIDB (http://cis.hku.hk/TISIDB/), which was a website for tumor immune interaction. The immune cell composition of DSC was analyzed using CIBERSORT (https://cibersort.stanford.edu/). R packages “ggplot2”, “ggpubr”, and “ggExtra” were used to process the data and calculated the correlation coefficient. |R|≥ 0.3 and *p* < 0.05 (*) were considered statistically significant.

### Co-expression genes of NFE2L3 in DSC

We used the packages “limma” and “BiocManager” to investigate the co-expressed genes and chemokine receptors that were both positively and negatively related to NFE2L3 expression in DSC.

### Protein–protein interaction network building

A protein–protein interaction (PPI) network was constructed using Search Tool for the Retrieval of Interacting Genes (STRING) (https://string-db.org/), with the following fixed principal parameters: a minimum required interaction score of 0.4, a maximum of 50 inter-actors to be displayed, and the sources of active interaction from experiments, text mining, and databases. The PPI network was visualized using Cytoscape (version 3.9.0).

### Gene ontology and kyoto encyclopedia of genes and genomes pathway enrichment analysis

Gene Ontology (GO) and Kyoto Encyclopedia of Genes and Genomes (KEGG) enrichment analyses and Genomes Enrichment Analyses (GSEA) were performed to assess the potential biological functions and pathways of NFE2L3 in DSC, using the packages “clusterProfiler”, “ggplot2” and “limma”.

### qRT-PCR

Total RNA extraction was extracted using the TRIzol Up Plus RNA Kit (Trnsgen). The cDNA synthesis of cDNA is done using M-MLV reverse transcription (Trnsgen). Quantitative reverse transcription polymerase chain reaction (qRT-PCR) was performed by SYBR Green PCR Master Mix (Takara).

The primer sequences of NFE2L3 for qRT-PCR were as follows:

forward, 5′-TTCAGCCAGGCTATAAGTCAGG-3′,

reverse, 5′-GCTCAGGATTGGTGGTATGAGA-3′.

The primer sequences of β-actin for qRT-PCR were as follows:

forward, 5′-CTCCATCCTGGCCTCGCTGT-3′,

reverse, 5′-GCTGTCACCGTTCC-3′.

### Cell line and culture

HGC-27 and AGS were purchased from the American Type Culture Collection (ATCC). Cells were cultured in RPMI-1640 containing 10% fetal bovine serum and cultured at 37℃ containing 5% CO_2_.

### siRNAs and plasmids transfection

Plasmids (pcDNA3.1) were purchased from Shanghai Genechem. Plasmids were transfected into AGS via Lipofectamine 2000 (ThermoFisher). siRNA (siNFE2L3#1, siNFE2L3#2, siNFE2L3#3) purchased from RIBOBIO and transfected into HGC-27 using Lipofectamine 2000. qRT-PCR was applied to detect the efficiency of NFE2L3 overexpression or knockdown.

The sequences of siRNA were as follows:

siNFE2L3#1 GCATGACCTTTATCATGAT

siNFE2L3#2 GTATGACCTTGACATAAAT

siNFE2L3#3 GTCAGGATGTGAATCTTCA

### Proliferation and migration assays

For proliferation experiments, approximately 1000 cells per well for each cell line were seeded in 96-well plates. The 450 nm absorbance was tested for 6 days (0, 1, 2, 3, 4, 5 days). The CCK-8 reagent was purchased from APExBIO (USA). We seeded 3 × 10^5^ cells per well in 6-well plate and collect images at 0 h, 12 h, and 24 h to calculate migration rates over time.

### Statistical analysis

The statistical analysis in this study was automatically calculated using the aforementioned online databases. *p* value < 0.05 was checked as the statistical significance. *p < 0.05, **p < 0.01, ***p < 0.001.

## Results

### NFE2L3 expression level in pan cancer

**Fig. 1:**
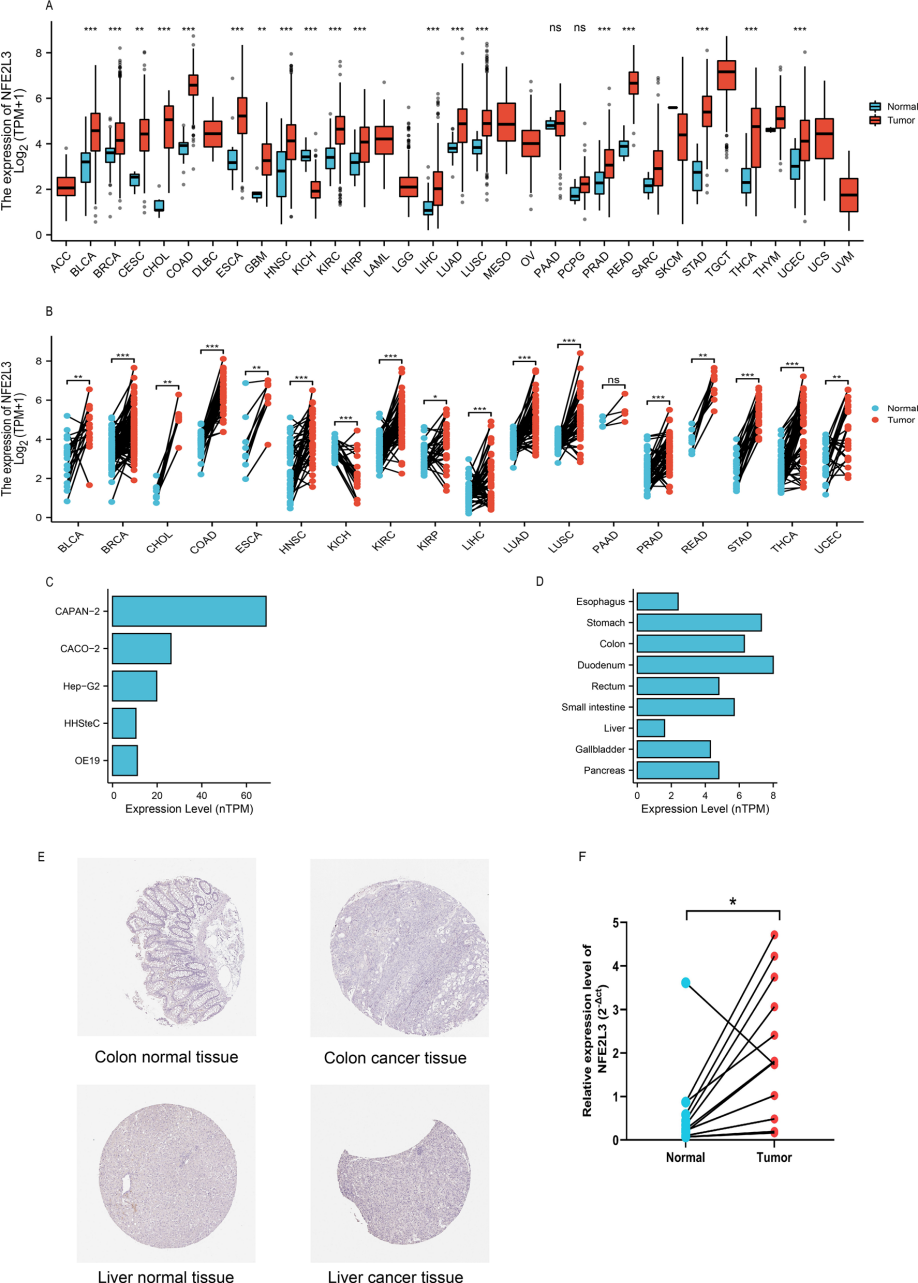
Expression level of NFE2L3 in pan cancer. **A** NFE2L3 is expressed at higher levels in pan-cancer unpaired tissues than in paracancerous tissues; **B** NFE2L3 is expressed at higher levels in pan-cancer paired tissues than in paracancerous tissues; **C** Expression levels of NFE2L3 in different cell lines from HPA dataset; **D** NFE2L3 expression in DSC tissues from HPA dataset; **E** Immunohistochemical staining results of liver cancer and colon cancer suggest that NFE2L3 is expressed at higher levels in tumor tissues than in normal tissues; **F** Validate the expression level of NFE2L3 in gastric cancer patients

To identify the expression level of NFE2L3 in cancers, a comparison is made to the normal tissue and cancer tissues. The results showed that NFE2L3 expression levels were elevated in DSC (Fig. 1A, B). Results were further validated by quantitative real-time PCR (qRT-PCR) in twelve STAD samples and its cancer adjacent tissues (Fig. 1F). The clinical characteristics of patients were presented in the supplemental materials (Table S14). The data from the Human Protein Atlas (HPA) annotate the abnormal expression of NFE2L3 in most tumor cell lines and cancer tissues (Fig. 1C, D, E).

### TMB and MSI of NFE2L3 in DSC

**Fig. 2:**
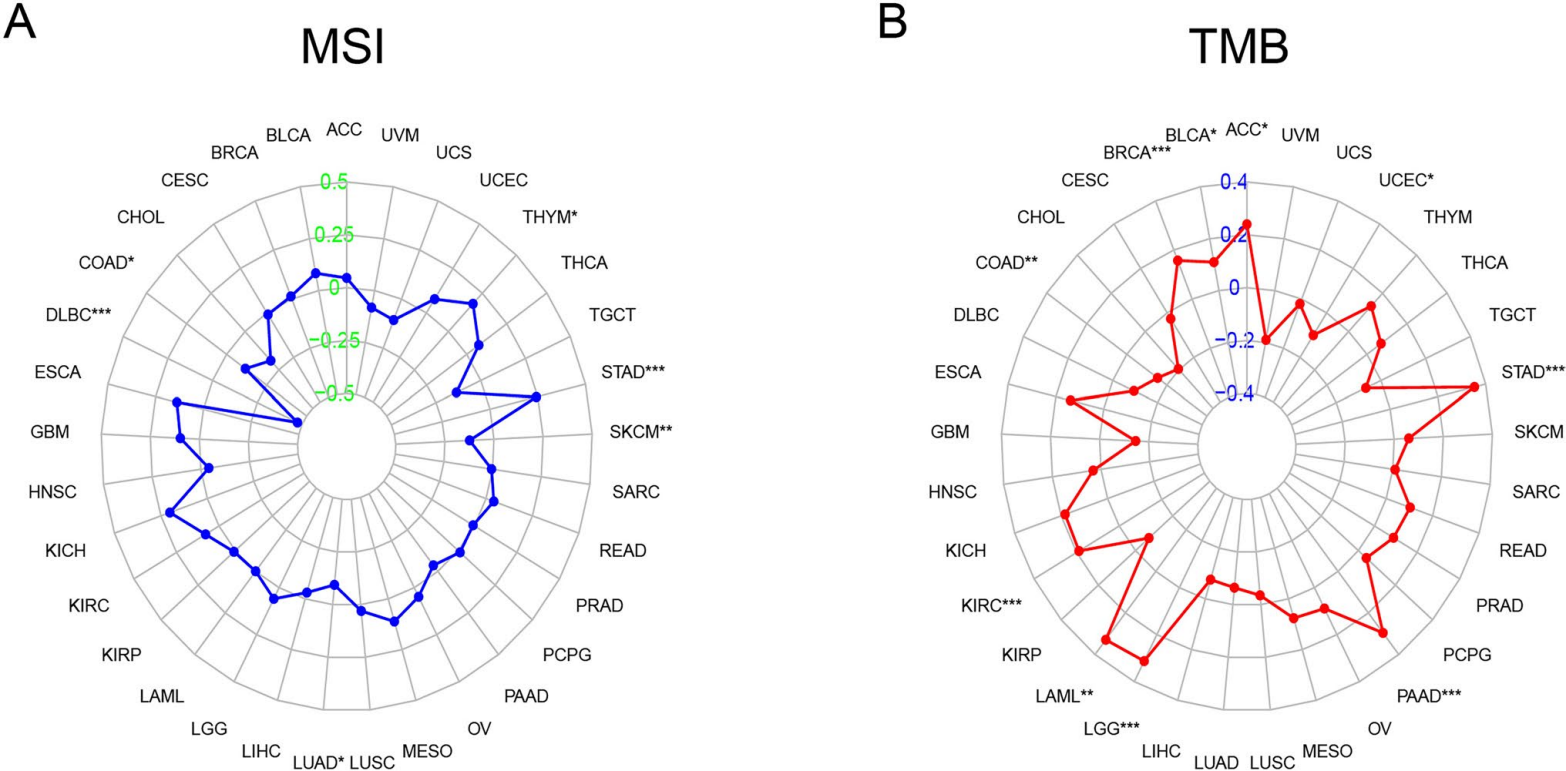
Relationship between NFE2L3 and MSI or TMB in DSC. **A** There was a close correlation between NFE2L3 and MSI in STAD, THYM, COAD, DLBC, SKCM, and LUAD; **B** There was a close correlation between NFE2L3 and TMB in ACC, BLCA, BRCA, LAML, LGG, PAAD, and STAD, COAD, KIRC, and UCEC

MSI were observed in STAD, THYM, COAD, DLBC, SKCM, and LUAD (Fig. 2A). TMB occurred in ACC, BLCA, BRCA, LAML, LGG, PAAD, and STAD, COAD, KIRC, and UCEC (Fig. 2B). The data implies that MSI burden and TMB levels are important hallmark of tumor response of DSC to immune checkpoint blockade. The *p* values and the relevant coefficients of MSI and TMB are shown in the supplementary materials (Table S1, S2).

### Diagnostic value of NFE2L3 in DSC

**Fig. 3:**
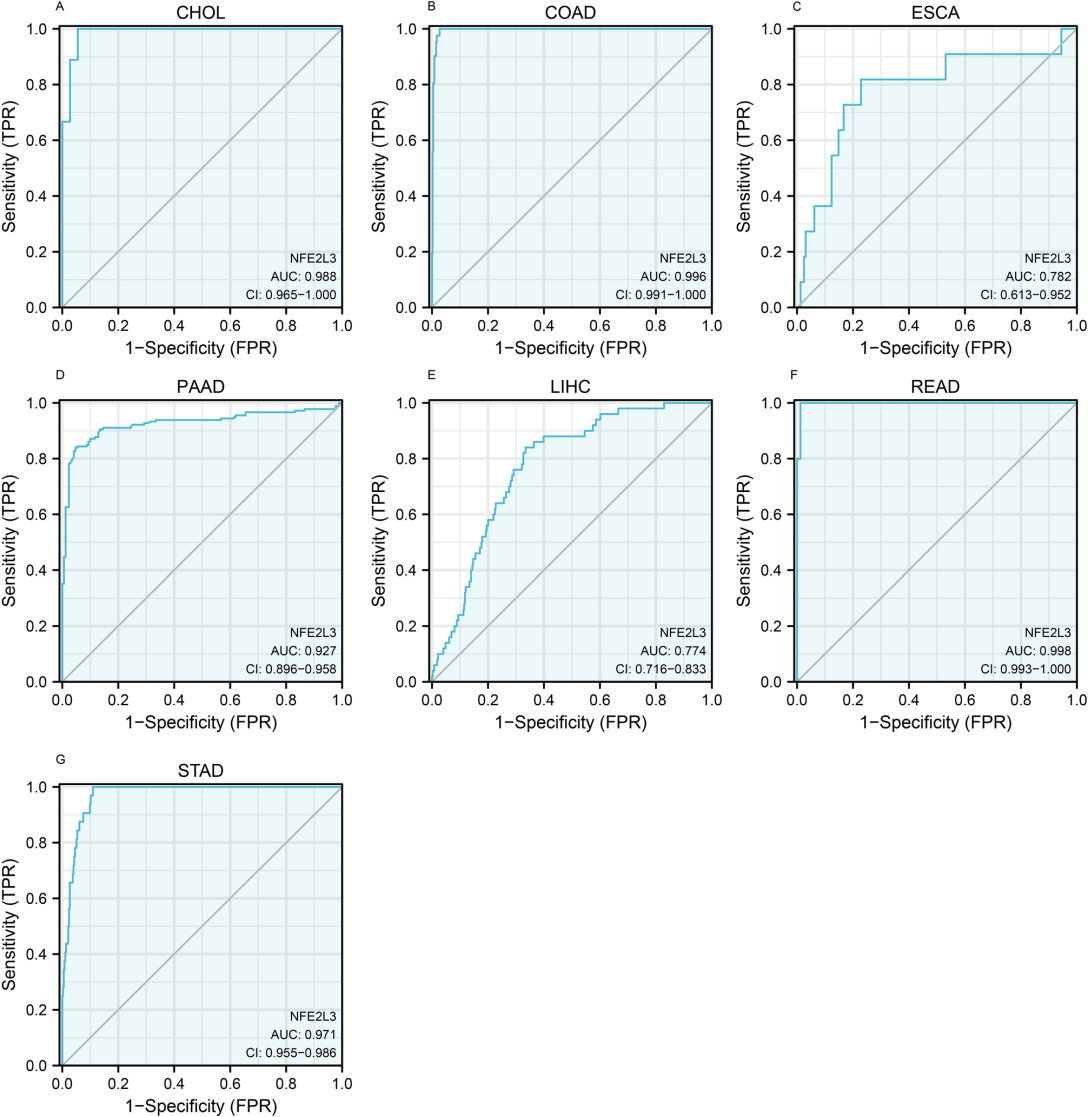
ROC curve of NFE2L3 in DSC. **A** ROC curve of NFE2L3 in CHOL; **B** ROC curve of NFE2L3 in COAD; **C** ROC curve of NFE2L3 in ESCA; **D** ROC curve of NFE2L3 in PAAD; **E** ROC curve of NFE2L3 in LIHC; **F** ROC curve of NFE2L3 in READ; **G** ROC curve of NFE2L3 in STAD

**Fig. 4:**
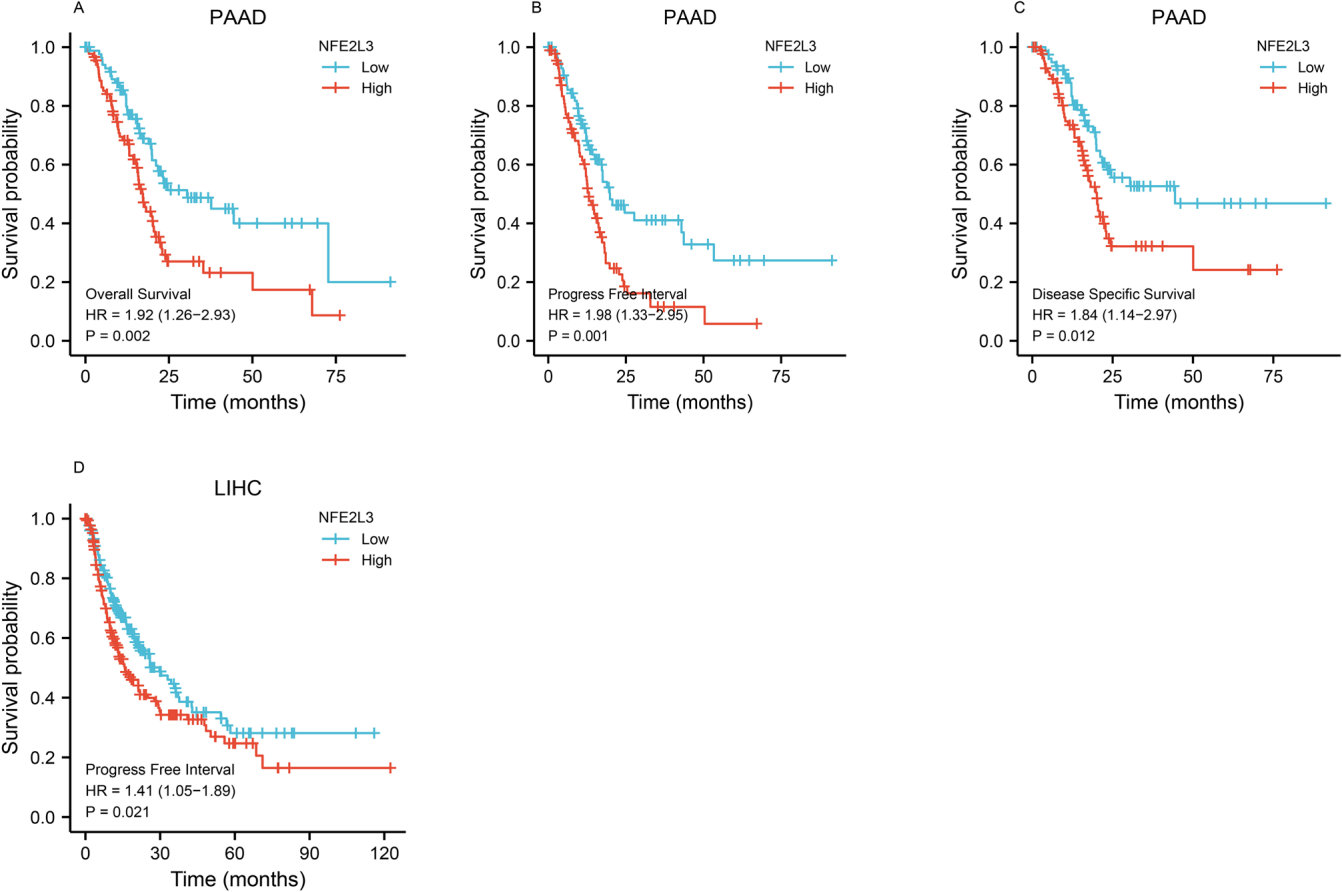
Prognostic value of NFE2L3 in DSC. **A** The overall survival is lower among PAAD patients with high-expression levels of NFE2L3; **B** The progress-free interval is lower among PAAD patients with high-expression levels of NFE2L3; **C** The disease-specific survival is lower among PAAD patients with high-expression levels of NFE2L3; **D** The progress-free interval rate is lower among LIHC patients with high-expression levels of NFE2L3

Based on the ROC curves, we concluded that NFE2L3 has a potential diagnostic value for DSC (Fig. 3, 4), particularly in STAD. The AUC values of ESCA, PAAD, LIHC, READ, and STAD were in the range of 0.87 to 1.00, indicating an ideal diagnostic value of NFE2L3 in the above-mentioned cancers.

### Prognostic value of NFE2L3 in DSC

The prognostic value of NFE2L3 in DSC was analyzed by comparing the overall survival (OS), progress-free interval (PFI), and disease-specific survival (DSS). In PAAD, high expression of NFE2L3 associated with poor OS, PFI, and DSS. Lower NFE2L3 expression was associated with higher PFI in LIHC. These findings indicate that NFE2L3 could be identified as a prognostic indicator.

### Clinical characteristics of NFE2L3 in DSC

To examine potential associations between clinical features and NFE2L3, we performed correlation analysis. There was a substantial correlation between NFE2L3 expression and both T (*p* = 0.006) and N (*p* = 0.032) stages in COAD. Age was substantially correlated with NFE2L3 expression in patients with ESCA (*p* = 0.012). In LIHC, NFE2L3 was strongly correlated with age (*p* = 0.034), T stage (*p* = 0.002), and pathological stage (*p* = 0.001). The pathogenic stage of PAAD was substantially correlated with NFE2L3 expression level (*p* = 0.017). There was a substantial correlation between pathological stage of STAD and NFE2L3 expression (*p* = 0.032). The clinical features of NFE2L3 in DSC are displayed in the supplemental materials (Tables S3–S9). Results presented above suggest that NFE2L3 has a close association with clinical characteristics of patients.

### NFE2L3 Expression in immune and molecular subtype

**Fig. 5:**
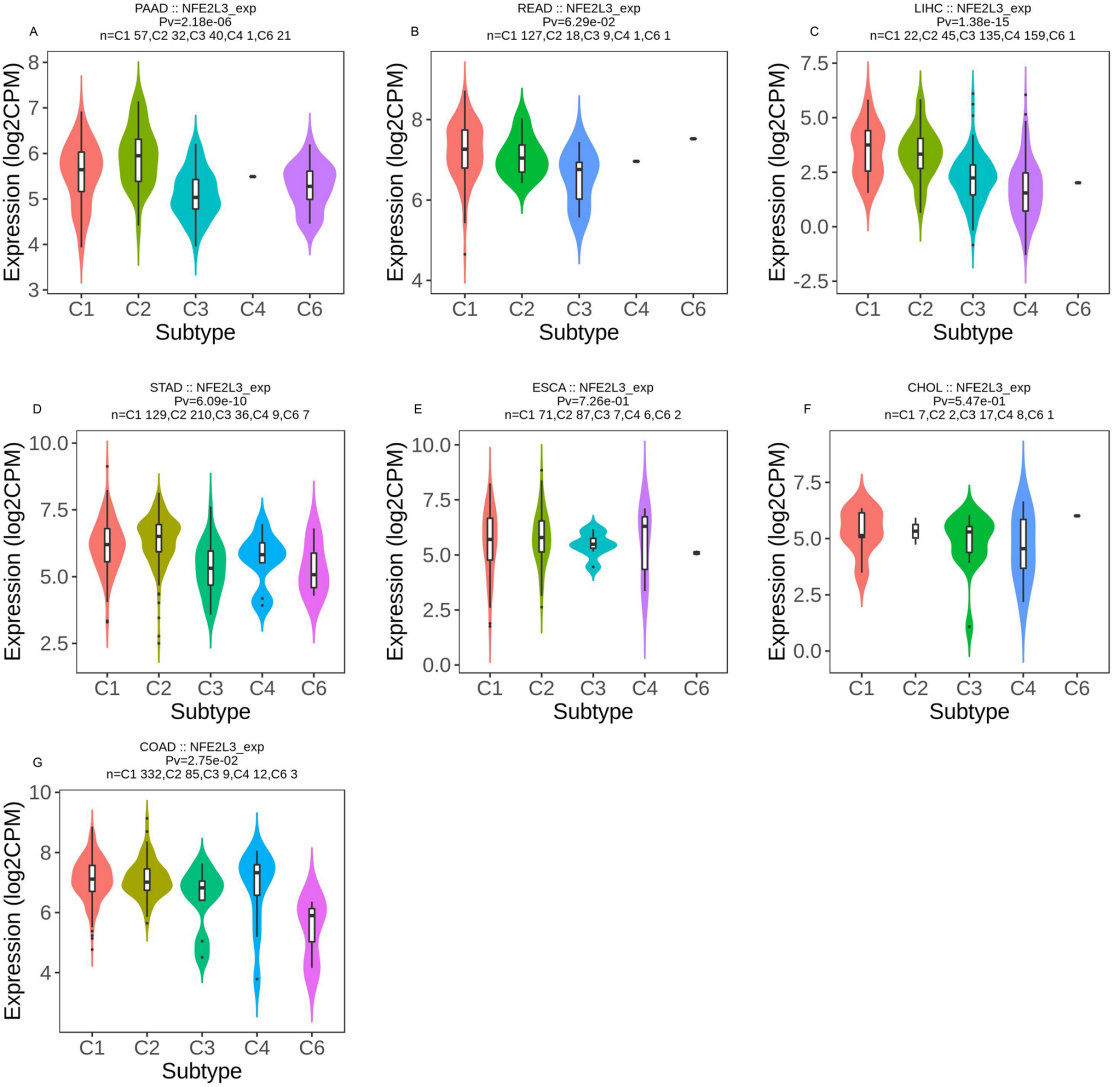
NFE2L3 expression in different immune subtypes. **A** Expression level of NFE2L3 of immune subtypes of PAAD; **B** Expression level of NFE2L3 of immune subtypes of READ; **C** Expression level of NFE2L3 of immune subtypes of LIHC; **D** Expression level of NFE2L3 of immune subtypes of STAD; **E** Expression level of NFE2L3 of immune subtypes of ESCA; **F** Expression level of NFE2L3 of immune subtypes of CHOL; **G** Expression level of NFE2L3 of immune subtypes of COAD

**Fig. 6:**
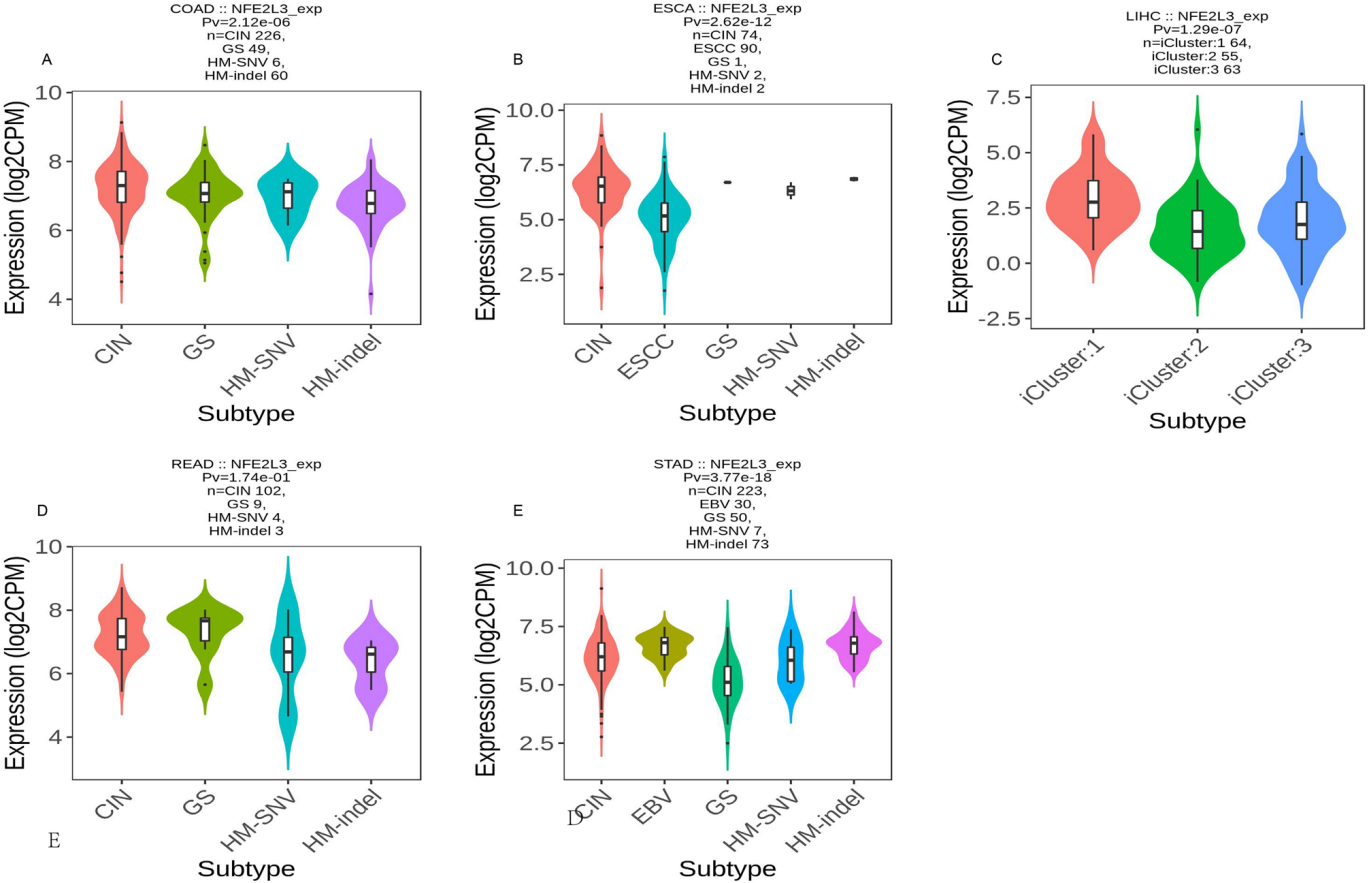
NFE2L3 expression level in different molecular subtypes. **A** In COAD, NFE2L3 expresses in four different molecular subtypes; **B** In ESCA, NFE2L3 expresses in CIN and ESCC; **C** In READ, NFE2L3 expresses in iCluster1, iCluster2, and iCluster3; **D** In LIHC, NFE2L3 expresses in four different molecular subtypes; **E** In STAD, NFE2L3 expresses in four different molecular subtypes

We observed both tumor immune (Fig. 5) and molecular subtypes (Fig. 6) of NFE2L3 in DSC. In READ, LIHC, and CHOL, NFE2L3 was predominantly expressed in the C1. In STAD and PAAD, NFE2L3 was expressed in the C2. NFE2L3 expressed in C4 in ESCA and COAD. In terms of the molecular subtype, NFE2L3 was expressed in the CIN of the COAD and ESCA. LIHC showed that NFE2L3 was mainly expressed in iCluster1. In READ, NFE2L3 was primarily expressed in the GS molecular subtype. In STAD, NFE2L3 was primarily expressed in the molecular subtype of the HM-indel. The results showed that NFE2L3 presents with multiple molecular and immune subtypes in DSC, thereby making NFE2L3 suitable targets for immunotherapy.

### Correlation between NFE2L3 expression levels and immune-related cell infiltration

**Fig. 7:**
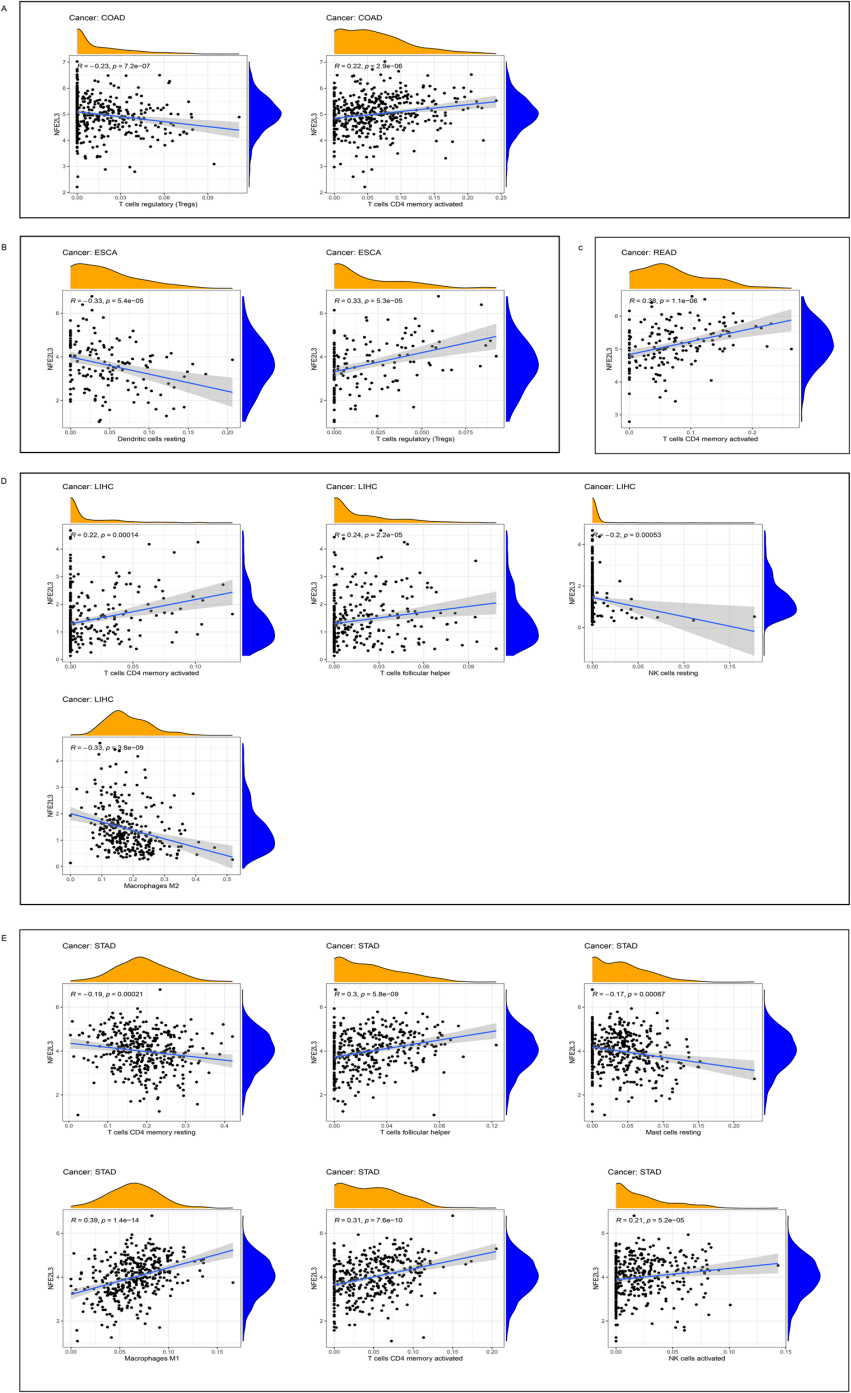
NFE2L3 expression in different immune cell infiltration. **A** In COAD, NFE2L3 is expressed in Tregs and CD4 memory cells; **B** In ESCA, NFE2L3 is expressed in two type immune cells; **C** In READ, NFE2L3 is expressed in CD4 memory cells; **D** In LIHC, NFE2L3 is expressed in four type immune cells; **E** In STAD, NFE2L3 is expressed in six type immune cells

We examined the relationship between NFE2L3 expression and immune-related cell infiltration in DSC (Fig. 7). In LIHC and STAD, we found strong correlations between NFE2L3 expression levels and different immune cells, especially in T regulatory cells (Tregs) and CD4^+^ T cells. This result suggested that there was close correlation between immune infiltration and NFE2L3 expression. NFE2L3 has the potential to be an important target for immunotherapy for DSC.

### NFE2L3 in immune-related genes and chemokine receptors

**Fig. 8:**
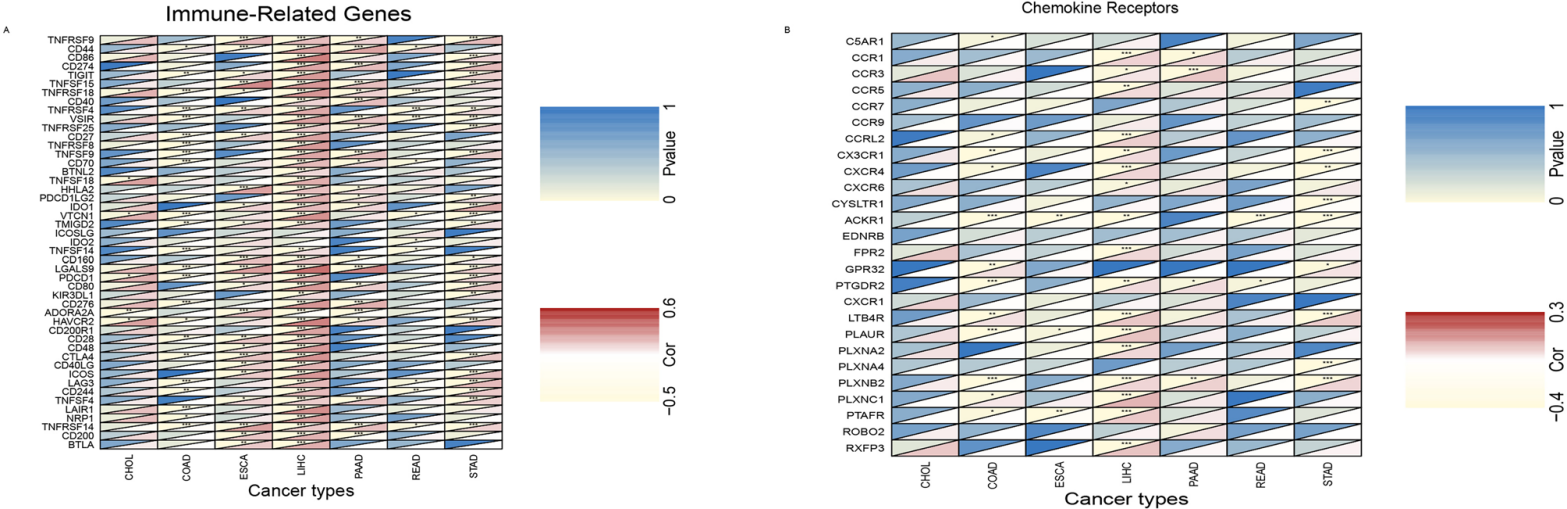
The relationships between NFE2L3 and immune-related genes or chemokine receptors. **A** Correlation between NFE2L3 and immune-related genes; **B** Correlation between NFE2L3 and Chemokine receptors

In this study, we aimed to determine the roles of NFE2L3 in immune-related genes (Fig. 8A) and chemokine receptors (Fig. 8B). The results showed that NFE2L3 presents a positive correlation with the majority of immune-related genes in LIHC, such as CD200 (*p* = 0.00, *R* = 0.339), CD274 (*p* = 0.00, *R* = 0.396), and CTLA4 (*p* = 0.00, *R* = 0.475). Many genes, such as TNFRSF9, CD274, CD44, TIGIT, TNFSF15, TNFRSF18, TNFRSF4, VISR, TNFSF9, IDO1, VTCN1, CD80, TNFRSF14, and CTLA4, exhibit significantly abnormal expression in most DSC. The correlation analysis of co-expression between NFE2L3 and chemokine receptors showed that numerous chemokine receptors, such as CX3CR1, CXCR4, ACKR1, PTGDR2, LTB4R, and PLXNB2, shared close co-expression relationships with NFE2L3 in most DSC, most notably in LIHC, COAD, and STAD. The related coefficients and the *p* value are reported in the supplemental materials (Tables S10–13).

### Proteins interact with NFE2L3

**Fig. 9:**
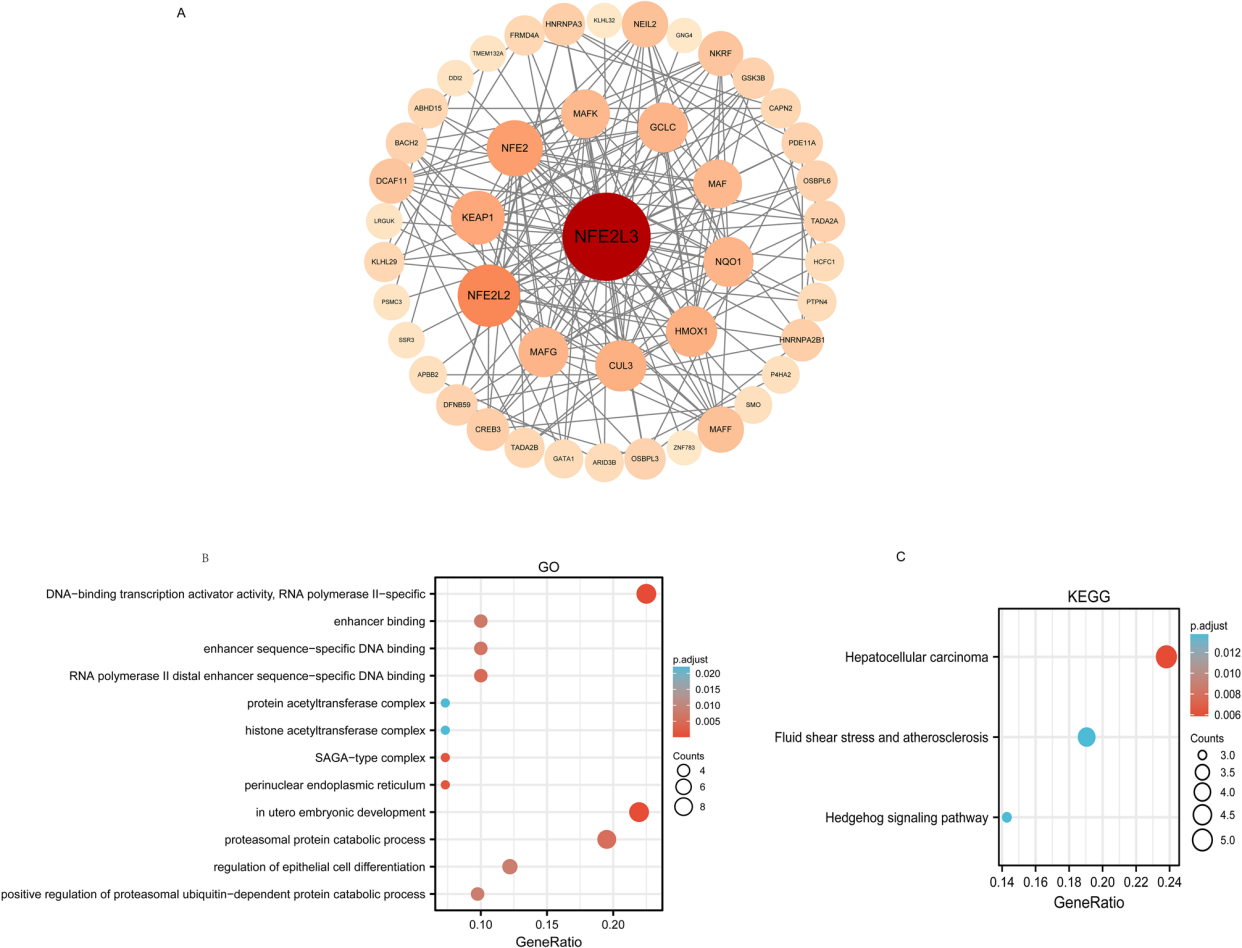
GO and KEGG analysis of proteins that interact with NFE2L3. **A** Proteins interact with NFE2L3; **B** Go analysis of interacting proteins; **C** KEGG analysis of interacting proteins

To obtain the potential target genes of NFE2L3, we conducted a PPI network. The results revealed that MAFK, NFE2, KEAP1, NFW2L2, MAFG, CUL3, HMOX1, NQO1, MAF, and GCLC are strong candidate target genes of NFE2L3. (Fig. 9A). To further interrogate the mechanism of NFE2L3 interaction, we examined its interaction through GO and KEGG analysis. The GO analysis results (Fig. 9B) showed that the activity of DNA-binding transcription, the specificity of RNA polymerase II, and the catabolic processes of proteasomal proteins were the major functions of the interactions. The KEGG analysis revealed that the protein pathways were mostly focused on the hedgehog signaling pathway (Fig. 9C).

### Gene set enrichment analysis

To examine the biological significance of NFE2L3 expression in DSC, we conducted GSEA. The TCGA tumor samples were categorized into two groups: high- and low-expression groups. The top GO terms and KEGG pathways were analyzed using GSEA. The GO annotation results indicated that NFE2L3 negatively regulated methylation in COAD and the metabolic processes in LIHC. In STAD, NFE2L3 expression affected signal transduction. The KEGG terms indicated that NFE2L3 activated autophagy in ESCA, whereas it inhibited drug metabolisms, such as P450, as well as calcium signaling pathways in READ and STAD. All GSEA data and images are displayed in the Supplementary Material.

### Biological functions of NFE2L3

**Fig. 10:**
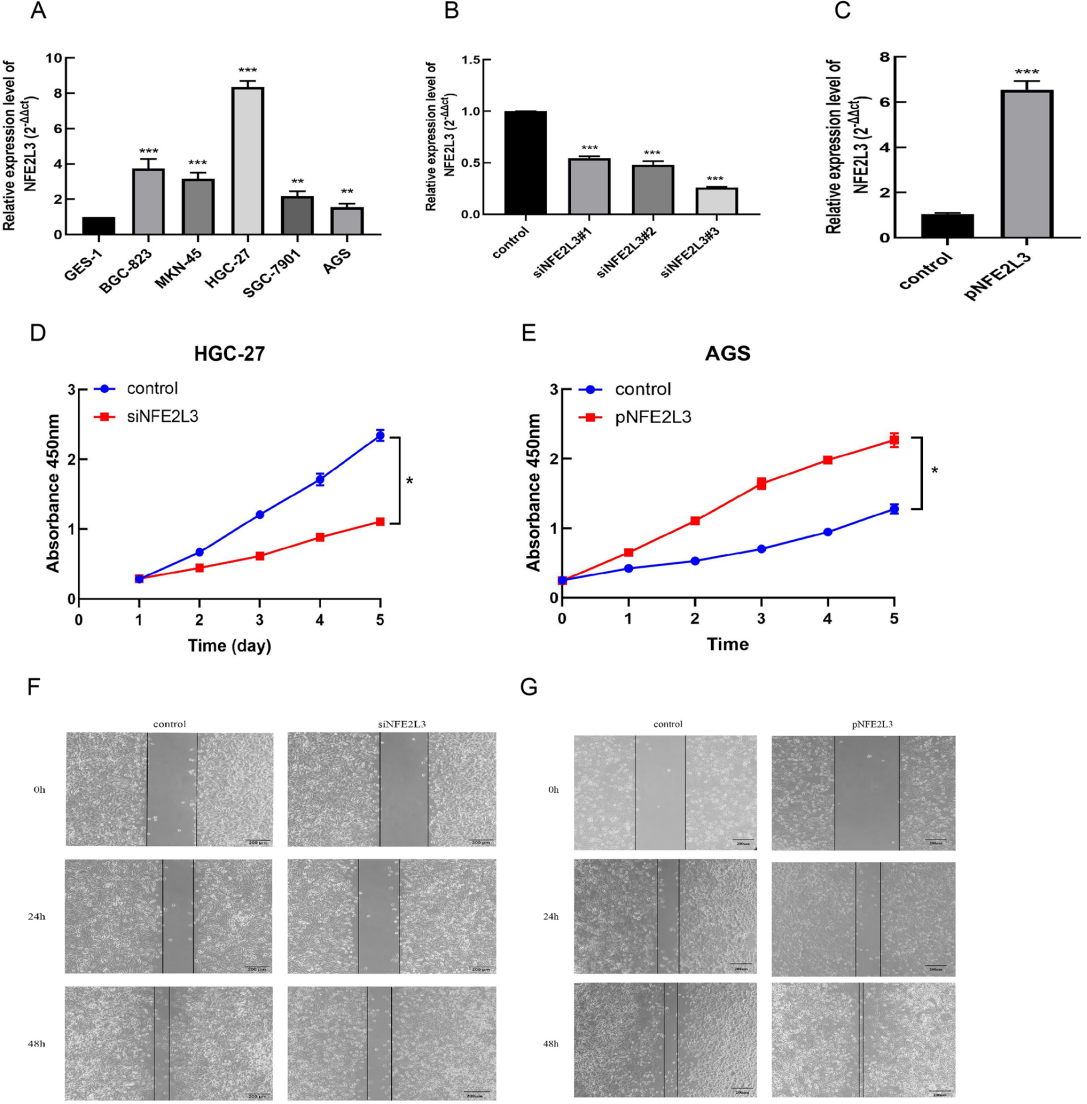
The biological function of NFE2L3 in gastric cancer. **A** The expression levels of NFE2L3 in cell lines; **B** Verification of knockdown efficiency of NFE2L3 in HGC-27; **C** Verification of overexpression efficiency of NFE2L3 in AGS; **D** The proliferation ability of NFE2L3 in HGC-27; **E** The proliferation ability of NFE2L3 in AGS; **F** The migration ability of NFE2L3 in HGC-27; **G** The migration ability of NFE2L3 in AGS

NFE2L3 expression level in gastric cancer cells was observed to be higher compared with normal gastric mucosal epithelial cells (Fig. 10A). Plasmids and siRNAs have an ideal efficacy in altering NFE2L3 expression levels (Fig. 10B, C). CCK-8 and wound healing assay showed that NFE2L3 knockdown suppressed the proliferation and migration of HGC-27. At the same time, overexpression of NFE2L3 promotes the cellular proliferation, the migration, and the invasion of AGS (Figs. 10D-G).

## Discussion

Our work in this study provides a comprehensive insight into the importance of NFE2L3 in DSC and revealed that NFE2L3 has great potential to become a widely utilized biomarker. NFE2L3 is found to be abnormally expressed in cancers and regulates DSC through multiple mechanisms. NFE2L3 has ideal clinical relevance for its good prognostic and diagnostic value.

Accumulating evidence suggests that NFE2L3 is crucial for the development of cancer. NFE2L3 belongs to the Cap ‘*n*’ Collar family, which plays a vital role in the cancer-promoting function by various mechanisms (Zhang et al. 2009; Sekine and Motohashi 2021). For example, NFE2L3 functions as an oncogene by triggering the Wnt/-catenin signaling pathway (Ren et al. 2020), absence of the NFE2L3 affects the formation and growth of tumors (Bury et al. 2019a, b). NFE2L3 regulates the immune microenvironment and has potential prognostic value in patients with kidney cancer (Feng et al. 2021). The absence of NFE2L3 results in significantly less inflammation in the colon, which inhibits an increase in the number and volume of tumors (Ju et al. 2020). Although the close relationship between NFE2L3 and cancers has been demonstrated, the clinical value and the underlying mechanism of NFE2L3 in cancers remain unclear.

A pan-cancer analysis showed that NFE2L3 is abnormally expressed in most malignancies, predominantly in the DSC. These findings have also been confirmed in cell lines and in cancer tissue data obtained from the HPA database. We further confirm this speculation in gastric cancer tissues and cell lines, the qRT-PCR results showed significant levels of NFE2L3. The above results lead to an important conclusion that NFE2L3 may involve in promoting tumorigenesis.

While prognostic clinical markers are useful, existing molecular makers are still of limited value in cancers. Recent studies suggested that NFE2L3 has an ideal diagnostic and prognostic value in bladder, colorectal, and renal cancers (Feng et al. 2021; Waku et al. 2020). Consistent with our findings, NFE2L3 functioned as an oncogene in PAAD and LIHC based on the fact that patients in the high-expression group had a worse prognosis than those in the low-expression group. Our results also confirmed that NFE2L3 showed ideal diagnostic and prognostic value for DSC. Therefore, NFE2L3 is expected to emerge as a novel indicator for the diagnosis and prognosis of DSC.

It is well established that cancer is a complex and heterogeneous genomic disease, and gene functions cannot be guaranteed based on the basis of standard definitions. One explanation for the aberrant function of genes in cancer is that different cellular contexts result in a wide range of pathological outcomes (Datta et al. 2020; Lin et al. 2020). A detailed mechanistic explanation for this problem has not yet been fully elucidated, and it is worth investigating in future.

ICIs as a mainstay of cancer treatment have provided novel strategies and improved clinical outcomes in multiple cancer types. However, response rates to immunotherapy vary due to several critical factors, including mutation burden, tumor-infiltrating lymphocytes, and regulatory checkpoint receptors (Carlino et al. 2021)_._ Researchers found that NFE2L3 played an important role in various immune-related pathways and immune cells. In Nfe2l3-/- mice, the levels of FOXP3 and the immune checkpoint markers cytotoxic T lymphocyte-associated protein 4 (CTLA4), TIM3, and lymphocyte activating 3 (LAG3) were increased (Bury et al. 2019a, b). The enrichment analyses revealed that NFE2L3 was associated with various immune-relevant pathways in kidney cancer and related to the infiltration ratios of 17 types of immune cells in patients with kidney cancer (Zhang et al. 2022). Nevertheless, the molecular mechanisms behind it remain mysterious.

The results of our study indicated a strong correlation between NFE2L3 expression and MIS in DSC. MSI is a certain phenomenon that often arises in tumors that have an underlying defect in DNA mismatch repair (Tomlinson et al. 2002). Patients with high MSI rates indicating poor median PFS or OS (De' Angelis et al. 2018; Ganesh et al. 2019; Asaoka et al. 2015). Therefore, it can be inferred that MSI could play a crucial role in shaping the progression of DSC. Relevant studies have indicated that cancers harbor high TMB means the potential to generate many neoantigens, making tumors more immunogenic for ICIs (Bravaccini et al. 2021; Chan et al. 2019). Expect MSI, the TMB also demonstrated the significance of the DSC. A higher TMB is normally related to an adverse outcome in advanced tumors.

According to our results, we revealed that NFE2L3 was closely correlated with immunity. It is expected to become novel immunotherapeutic target for DSC in future. Further studies should continue to investigate the mechanisms of NFE2L3 in immunotherapy.

Our findings indicated a significant positive correlation between NFE2L3 and immune or molecular subtypes. Cellular and immune subtypes are the main factors responsible for tumor heterogeneity. Treatment elicits varied responses from tumors, owing to the genetic diversity within the tumor. The combination of immune and molecular subtypes for precisely targeted tumor therapy will also greatly enhance the current status of immunotherapeutic efficacy. Therefore, we believe that NFE2L3 plays a vital role in DSC immunotherapy. Therapeutic cancer vaccines remain a valid immunotherapy option and hold significant implications for the clinical advancement of cancer patients (Pe'er et al. 2021; Baharom et al. 2022). Over the past few years, there has been a significant amount of research conducted on vaccines and therapeutics specifically designed for the innate and adaptive immune systems in the field of cancer treatment (Peng et al. 2022; Ratnam et al. 2019). Thus, the important functions of immune cells in tumor-specific immunity against immune cells can be understood. Our findings showed that compared with non-responders, patients who generated antibody responses to therapy exhibited prolonged DFS. We have reason to believe that NFE2L3 to be a potential target for immunotherapy in future.

A multitude of studies have indicated that patients with immune cell infiltration tumors exhibit a more favorable response to ICI than patients with non-immunological tumors. Recent research has revealed that NFE2L3 plays a pivotal role in cancer development (Wang et al. 2018; Zhang et al. 2019), but the precise mechanisms have yet to be elucidated. The findings of this research indicated that DSCs experienced immune cell infiltration. The immune cell infiltration encompasses a wide range of cell types, such as Tregs, T cells, dendritic cells, NK cells, T follicular helper cells, macrophages, mast cells, and monocytes. In STAD, we observed a strong correlation between NFE2L3 expression and immune cells abundance. The research revealing the correlation of immune cell infiltration with NFE2L3 may open unique avenues for precise cancer therapeutics against cancers.

Co-expression analysis can be employed as a means to identify disease-associated genes and gene functions in tumors. Chemokines combine with the cell surface chemokine receptors to perform biological functions, such as the chemotaxis, leukocyte migration, and inflammatory activities (Charo and Ransohoff 2006; Arimont et al. 2017). The causative role of chemokines is variable, and they play an important role in many diseases, such as cancer, viral infections, inflammatory, and autoimmune diseases. Researchers have found that chemokines affect anti-tumorigenic activity by regulating tumor angiogenesis and infiltration of immune cells (Lacalle et al. 2017). The results of our study showed a strong correlation between NFE2L3 and immune-related genes or chemokine receptors. In COAD, LIHC, and STAD, there was a close correlation between NFE2L3 expression and the chemokine receptors, such as C-X3-C motif chemokine receptor 1 (CX3CR1), plexin B2 (PLXNB2), atypical chemokine receptor 1 (ACKR1), and formyl peptide receptor 2 (FPR2). Anja Schmall found that the tumor-associated crosstalk between macrophages and cancer cells via the CCR2 and CX3CR1 signaling pathways directed the lung cancer growth and metastasis (Schmall et al. 2015). Circ_0013958 plays an oncogenic role in ovarian cancer by regulating the miR-637/PLXNB2 axis (Liang et al. 2021). Immune-related genes, such as PD-1, PD-L1, and CTLA4, have been successfully used in tumor immunotherapy and have been shown to have a significant effect on B cell lymphomas, HCC, and non-small cell lung cancer (Xu-Monette and Zhou 2018; Kamada et al. 2019), (Thommen et al. 2018). This study revealed novel immune-related genes that are co-expressed with NFE2L3 in DSC, including TIGIT, IDO1, ADORA2A, and CD70, which can be used as immune therapy, checkpoints, and biomarkers (Zhai et al. 2018; Chauvin and Zarour 2020). Therefore, combining immune-related genes or chemokine receptors with NFE2L3 may enhance the efficacy of DSC diagnosis and immunotherapy.

We conducted GO enrichment and KEGG analysis on the NFE2L3 binding proteins, we found that the Hedgehog signaling pathway is the primary target of protein catabolic processes and cell differentiation. Hedgehog signaling pathway activated tumors by driving EMT, and the inhibitor of Hedgehog signaling pathway exhibited remarkable clinical outcomes in various types of cancer Zhang et al. (2009). Nevertheless, the investigation into the differential regulation of target genes by NFE2L3 remains to be investigated.

NFE2L3 is involved in the regulation of various biological and cellular processes, such as cell cycle, cell differentiation, or inflammatory processes. We performed GSEA and found that NFE2L3 was significantly associated with epigenetics, methylation modification, cell cycle regulation, calcium signaling pathway and steroid hormone biosynthesis. Consequently, the distinct manifestation of NFE2L3 assumes a crucial function in DSC through various biological functions and signaling pathways, ultimately impacting the process of tumorigenesis. Nevertheless, a comprehensive understanding of the findings would require further experimental validation.

To validate the bioinformatics findings, in vivo experiments conducted on gastric cancer cells demonstrated the role of NFE2L3 as an oncogene, facilitating the proliferation and movement of gastric cancer cells. NFE2L3 enhances tumor cell migration ability by affecting the EMT through Wnt/β-catenin signaling pathway (Ren et al. 2020; Zeng and Ju 2018). These findings and our hypothesis are validated experimental results. More in vitro research is needed to gain a better understanding of how things work in future.

The outcomes of these bioinformatics analyses lay the groundwork for future comprehensive investigations into the mechanisms underlying tumorigenesis and evolution. NFE2L3 was found to be aberrantly expressed in DSC, and significantly correlated with the diagnosis and prognosis of DSC patients. In addition, in vivo results suggest that NFE2L3 may act as an oncogene roles in gastric cancer through promoting the gastric cancer cells growth and migration.

## Conclusions

In conclusion, we identified a specific role of NFE2L3 in DSC. The results of our study indicated that NFE2L3 was differentially expressed in cancers and closely related to clinical features. Therefore, it has the potential prognostic and diagnostic values for DSC. In addition, NFE2L3 was closely related to immune filtration, immune subtype, and molecular subtype and co-expressed with numerous genes and chemokines. These findings suggest that NFE2L3 has the potential to emerge as a novel therapeutic target for DSC. Additional experimental studies are required to verify these findings.

## Data Availability

All relevant data are within the manuscript and its Additional files.

## References

[CR1] Arimont M, Sun SL, Leurs R, Smit M, de Esch IJP, de Graaf C (2017) Structural analysis of chemokine receptor-ligand interactions. J Med Chem 60:4735–4779 28165741 10.1021/acs.jmedchem.6b01309 PMC5483895

[CR2] Asaoka Y, Ijichi H, Koike K (2015) PD-1 blockade in tumors with mismatch repair deficiency. N Engl J Med 373:1979. 10.1056/NEJMc1510353 26559583 10.1056/NEJMc1510353

[CR3] Baharom F, Ramirez-Valdez RA, Khalilnezhad A, Khalilnezhad S, Dillon M, Hermans D (2022) Systemic vaccination induces CD8(+) T cells and remodels the tumor microenvironment. Cell 185:4317–4332. 10.1016/j.cell.2022.10.006 36302380 10.1016/j.cell.2022.10.006 PMC9669246

[CR4] Bravaccini S, Bronte G, Ulivi P (2021) TMB in NSCLC: a broken dream? Int J Mol Sci 22:6356. 10.3390/ijms22126536 34207126 10.3390/ijms22126536 PMC8234326

[CR5] Bury M, Le Calvé B, Lessard F, Dal Maso T, Saliba J, Michiels C, Gerardo F, Blank V (2019a) NFE2L3 controls colon cancer cell growth through regulation of DUX4, a CDK1 inhibitor. Cell Rep 29:1469–1481. 10.1016/j.celrep.2019.09.087 31693889 10.1016/j.celrep.2019.09.087

[CR6] Bury M, Le Calvé B, Lessard F, Dal Maso T, Saliba J, Michiels C, Ferbyre G, Blank V (2019b) NFE2L3 controls colon cancer cell growth through regulation of DUX4, a CDK1 Inhibitor. Cell Rep 29(6):1469–1481. 10.1016/j.celrep.2019.09.087 31693889 10.1016/j.celrep.2019.09.087

[CR7] Carlino MS, Larkin J, Long GV (2021) Immune checkpoint inhibitors in melanoma. Lancet 398:1002–1014. 10.1016/S0140-6736(21)01206-X 34509219 10.1016/S0140-6736(21)01206-X

[CR8] Chan TA, Yarchoan M, Jaffee E, Swanton C, Quezada SA, Stenzinger A, Perters S (2019) Development of tumor mutation burden as an immunotherapy biomarker: utility for the oncology clinic. Ann Oncol 30:44–56. 10.1093/annonc/mdy495 30395155 10.1093/annonc/mdy495 PMC6336005

[CR9] Charo IF, Ransohoff RM (2006) The many roles of chemokines and chemokine receptors in inflammation. N Engl J Med 354:610–621. 10.1056/NEJMra052723 16467548 10.1056/NEJMra052723

[CR10] Chauvin JM, Zarour HM (2020) TIGIT in cancer immunotherapy. J Immunother Cancer 8:e000957. 10.1136/jitc-2020-000957 32900861 10.1136/jitc-2020-000957 PMC7477968

[CR11] Chen LB, Lu DW, Sun KK, Xu YM, Hu PP, Li XP (2019) Identification of biomarkers associated with diagnosis and prognosis of colorectal cancer patients based on integrated bioinformatics analysis. Gene 692:119–125. 10.1016/j.gene.2019.01.001 30654001 10.1016/j.gene.2019.01.001

[CR12] Chevillard G, Blank V (2011) NFE2L3 (NRF3): the cinderella of the cap’n’collar transcription factors. Cell Mol Life Sci 68:3337–3348. 10.1007/s00018-011-0747-x 21687990 10.1007/s00018-011-0747-x PMC11114735

[CR13] Datta N, Chakraborty S, Basu M, Ghosh MK (2020) Tumor suppressors having oncogenic functions: the double agents cells. Cells 10:46. 10.3390/cells10010046 33396222 10.3390/cells10010046 PMC7824251

[CR14] De Angelis GL, Bottarelli L, Azzoni C, De Angelis N, Leandro G, Di Mario F, Gaiani F, Negri F (2018). Microsatellite instability in colorectal cancer. Acta Biomed, doi: 10.23750/abm.v89i9-S.7960 10.23750/abm.v89i9-S.7960 PMC6502181 30561401

[CR15] Feng M, Zhao Z, Yang M, Ji J, Zhu D (2021) T-cell-based immunotherapy in colorectal cancer. Cancer Lett 498:201–209. 10.1016/j.canlet.2020.10.040 33129958 10.1016/j.canlet.2020.10.040

[CR16] Galluzzi L, Humeau J, Buqué A, Zitvogel L, Kroemer G (2020) Immunostimulation with chemotherapy in the era of immune checkpoint inhibitors. Nat Rev Clin Oncol 17:725–741. 10.1038/s41571-020-0413-z 32760014 10.1038/s41571-020-0413-z

[CR17] Ganesh K, Stadler ZK, Cercek A, Mendelsohn RB, Shia J, Segal NH, Diaz LA Jr (2019) Immunotherapy in colorectal cancer: rationale, challenges and potential. Nat Rev Gastroenterol Hepatol 16:361–375. 10.1038/s41575-019-0126-x 30886395 10.1038/s41575-019-0126-x PMC7295073

[CR18] Ju Q, Li XM, Zhang H, Yan S, Li Y, Zhao YJ (2020) NFE2L2 is a potential prognostic biomarker and is correlated with immune infiltration in brain lower grade glioma: a pan-cancer analysis. Oxid Med Cell Longev 2020:3580719. 10.1155/2020/3580719 33101586 10.1155/2020/3580719 PMC7569466

[CR19] Kamada TH, Togashi YK, Tay C, Ha D, Sasaki A, Nakamura Y, Sato E, Fukuoka S, Tada Y, Tanaka A, Morikawa H, Kawazoe A, Kinoshita T, Shitara K, Skaguchi S, Nishikawa H (2019) PD-1(+) regulatory T cells amplified by PD-1 blockade promote hyperprogression of cancer. Proc Natl Acad Sci USA 116:9999–10008. 10.1073/pnas.1822001116 31028147 10.1073/pnas.1822001116 PMC6525547

[CR20] Lacalle RA, Blanco R, Carmona-Rodríguez L, Martín-Leal A, Mira E, Mañes S (2017) Chemokine receptor signaling and the hallmarks of cancer. Int Rev Cell Mol Biol 331:181–244. 10.1016/bs.ircmb.2016.09.011 28325212 10.1016/bs.ircmb.2016.09.011

[CR21] Liang YF, Meng KY, Qiu R (2021) Circular RNA Circ_0013958 functions as a tumor promoter in ovarian cancer by regulating miR-637/PLXNB2 axis. Front Genet 12:644451. 10.3389/fgene.2021.644451 34367233 10.3389/fgene.2021.644451 PMC8334736

[CR22] Lin A, Zhang J, Luo P (2020) Crosstalk between the MSI status and tumor microenvironment in colorectal cancer. Front Immunol 2020(11):2039. 10.3389/fimmu.2020.02039 10.3389/fimmu.2020.02039 PMC7435056 32903444

[CR23] Pe’er D, Ogawa S, Elhanani O, Keren L, Oliver TG, Wedge D (2021) Tumor heterogeneity. Cancer Cell 39:1015–1017. 10.1016/j.ccell.2021.07.009 34375606 10.1016/j.ccell.2021.07.009

[CR24] Peng S, Chen S, Hu W, Mei J, Zeng XZ, Su TH, Wang W, Chen ZB, Xiao H, Zhou Q, Li B, Xie YB, Hu HJ, He MH, Han YY, Tang LQ, Ma YF, Li XH, Zhou XJ, Dan ZH, Liu ZL, Tan JH, Xu LX (2022) Combination neoantigen-based dendritic cell vaccination and adoptive t-cell transfer induces antitumor responses against recurrence of hepatocellular carcinoma. Cancer Immunol Res 10:728–744. 10.1158/2326-6066.CIR-21-0931 35476700 10.1158/2326-6066.CIR-21-0931

[CR25] Qian JQ, Huang C, Zhu Z, He YH, Wang Y, Feng N, He SM, Li XS, Zhou LQ, Zhang CJ, Gong YQ (2022) NFE2L3 promotes tumor progression and predicts a poor prognosis of bladder cancer. Carcinogenesis 43:457–468. 10.1093/carcin/bgac006 35022660 10.1093/carcin/bgac006

[CR26] Ratnam NM, Gilbert MR, Giles AJ (2019) Immunotherapy in CNS cancers: the role of immune cell trafficking. Neuro Oncol 21:37–46. 10.1093/neuonc/noy084 29771386 10.1093/neuonc/noy084 PMC6303437

[CR27] Ren YG, Wang YJ, Hao S, Yang YH, Xiong WD, Qiu L, Tao J, Tang A (2020) NFE2L3 promotes malignant behavior and EMT of human hepatocellular carcinoma (HepG2) cells via Wnt/β-catenin pathway. J Cancer 11:6939–6949. 10.7150/jca.48100 33123284 10.7150/jca.48100 PMC7591994

[CR28] Saliba J, Coutaud B, Makhani K, Epstein Roth N, Jackson J, Park JY, Gagnon N, Costa P, Jeyakumar T, Bury M, Beauchemin N (2022) Loss of NFE2L3 protects against inflammation-induced colorectal cancer through modulation of the tumor microenvironment. Oncogene 41:1563–1575. 10.1038/s41388-022-02192-2 35091681 10.1038/s41388-022-02192-2 PMC8913363

[CR29] Schmall A, Al-Tamari HM, Herold S, Kampschulte M, Weigert A, Wietelmann A, Vipotnik N, Grimminger F, Seeger W, Pullamsetti SS, Savai R (2015) Macrophage and cancer cell cross-talk via CCR2 and CX3CR1 is a fundamental mechanism driving lung cancer. Am J Respir Crit Care Med 191:437–447. 10.1164/rccm.201406-1137OC 25536148 10.1164/rccm.201406-1137OC

[CR30] Sekine H, Motohashi H (2021) Roles of CNC transcription factors NRF1 and NRF2 in cancer. Cancers (basel) 13:541. 10.3390/cancers13030541 33535386 10.3390/cancers13030541 PMC7867063

[CR31] Siegel RL, Miller KD, Fuchs HE, Jemal A (2021) Cancer statistics, 2021. CA Cancer J Clin 71:7–33. 10.3322/caac.21654 33433946 10.3322/caac.21654

[CR32] Thommen DS, Koelzer VH, Herzig P, Roller A, Trefny M, Dimeloe S, Kiialainen A, Hanhar J, Schill C, Hess C, Princetrans SS, Wiese M, Lardinois D, Ho PC, Klein C, Karanikas V, Mertz KD, Schumacher TN, Zippelius A (2018) Tcriptionally and functionally distinct PD-1(+) CD8(+) T cell pool with predictive potential in non-small-cell lung cancer treated with PD-1 blockade. Nat Med 24:994–1004. 10.1038/s41591-018-0057-z 29892065 10.1038/s41591-018-0057-z PMC6110381

[CR33] Tomlinson I, Halford S, Aaltonen L, Hawkins N, Ward R (2002) Does MSI-low exist? J Pathol 197:6–13. 10.1002/path.1071 12081205 10.1002/path.1071

[CR34] Waku T, Katayama H, Hiraoka M, Hatanaka A, Nakamura N, Tanaka Y, Tamura N, Wantanabe A, Kobayashi A (2020) NFE2L1 and NFE2L3 Complementarily maintain basal proteasome activity in cancer cells through CPEB3-mediated translational repression. Mol Cell Biol 40:e00010–e00020. 10.1128/MCB.00010-20 32366381 10.1128/MCB.00010-20 PMC7324849

[CR35] Wang H, Zhan M, Yang RM, Shi YH, Liu Q (2018) Wang J. Elevated expression of NFE2L3 predicts the poor prognosis of pancreatic cancer patients. Cell Cycle 17:2164–2174. 10.1080/15384101.2018.1520558/30196752 10.1080/15384101.2018.1520558 PMC6226237

[CR36] Wang JX, Choi SYC, Niu X, Kang N, Xue H, Killam J, Yuzhou W (2020) Lactic Acid and an acidic tumor microenvironment suppress anticancer immunity. Int J Mol Sci 2020:21. 10.3390/ijms21218363 10.3390/ijms21218363 PMC7664620 33171818

[CR37] Xie FF, You YL, Huang JH, Guan C, Chen ZJ, Fang M, Yao F (2021) Association between physical activity and digestive-system cancer: an updated systematic review and meta-analysis. J Sport Health Sci 10:4–13. 10.1016/j.jshs.2020.09.009 33010525 10.1016/j.jshs.2020.09.009 PMC7856558

[CR38] Xu-Monette ZY, Zhou JF (2018) Young KH. PD-1 expression and clinical PD-1 blockade in B-cell lymphomas. Blood 131:68–83. 10.1182/blood-2017-07-740993 29118007 10.1182/blood-2017-07-740993 PMC5755041

[CR39] Zeng X, Ju DW (2018) Hedgehog signaling pathway and autophagy in cancer. Int J Mol Sci 19:2279. 10.3390/ijms19082279 30081498 10.3390/ijms19082279 PMC6121518

[CR40] Zhai L, Ladomersky E, Lenzen A, Nguyen B, Patel R, Lauing KL, Wu MJ, Wainwright DA (2018) IDO1 in cancer: a gemini of immune checkpoints. Cell Mol Immunol 15:447–457. 10.1038/cmi.2017.143 29375124 10.1038/cmi.2017.143 PMC6068130

[CR41] Zhang YG, Kobayashi A, Yamamoto MK, Hayes JD (2009) The Nrf3 transcription factor is a membrane-bound glycoprotein targeted to the endoplasmic reticulum through its N-terminal homology box 1 sequence. J Biol Chem 284:3195–3210. 10.1074/jbc.M805337200 19047052 10.1074/jbc.M805337200

[CR42] Zhang LH, Hu DL, Tang B, Cheng Y, Jiao C, Cheng L, Tan ZR, Zhou HH (2019) NFE2L3 Inhibition Induces Cell Cycle Arrest at the G0/G1 Phase in colorectal cancer cells through downregulating CCND1 and pRb1-ser807/811. Dis Markers. 10.1155/2019/2829798 31191746 10.1155/2019/2829798 PMC6525936

[CR43] Zhang Q, Tang D, Zha A, He JQ, Li DD, Chen YM, Cai WX, Dai J, Luan SD, Yin LH, Zhang W, Dai Y (2022) NFE2L3 as a potential functional gene regulating immune microenvironment in human kidney cancer. Biomed Res Int 2022:9085186. 10.1155/2022/9085186 36337840 10.1155/2022/9085186 PMC9629961

